# Third Set of Teeth

**Published:** 1896-06

**Authors:** 


### THIRD SET OF TEETH.

The Sunday *Herald* of Syracuse, New York, reports a case at considerable length of one James Slattery, of that city, who has apparently erupted late in life a portion of the full denture with the prospect of more teeth presenting in the near future. Dr. S. B. Palmer, of Syracuse, made an examination of the case and reports as follows:

"In the inferior maxilla are eight teeth located as follows: four incisors and two cuspidati corresponding with the cuspidati of the superior maxilla, and two small bicuspids on the right side. The cuspid on the left side and the second bicuspid on the right side are somewhat loose. The alveolar process does not extend up and entirely around the roots. The other teeth are firm and of usual size and length, but are somewhat overlapped from crowding and discolored by smoking; in other respects they present the appearance one would expect at his age.

"On the left side, in place of the two posterior teeth, ordinarily found, are three parts of teeth, resembling roots, having grown up partially out of the gums. They are not firm or well-developed teeth. The man insists that they also appeared within the time mentioned, two years previously, and that they belong to the same set. One is loose and turned against the cheek, and will require extraction. The other portions of the jaw show no signs of further eruption of teeth, as considerable absorption has taken place.

"An examination of the superior maxilla shows that the third set, more numerous than those in the inferior, will soon make their appearance. The jaw is thick and full nearly all the way back, raising the lip and giving the fulness peculiar to a child's jaw of six or seven years, or about the period of the eruption of the teeth of the permanent set."

The history of this singular case is briefly as follows: James Slattery is a well developed man of six feet two inches and weighing two hundred and thirty pounds. At the age of eighty-seven he was edentulous, having lost his second set of teeth. At about this period he noticed a painful condition of the gums, or, as he expressed it, "they began to ache badly," and for two years they continued to erupt in the inferior maxilla, and will, in time, develop in the superior.

This seems the best attested case of third dentition we have met with on record, and it is hoped Dr. Palmer will secure testimony as to the character of the set prior to becoming edentulous through advancing years. The dental profession has regarded a third set of teeth as a myth, and with some reason, as most of the so-called third sets have proved, on investigation, to be simply delayed dentition of the regular second set.—[Ed.]

